# Integrative Medicine Approaches: Bridging the Gap Between Conventional and Renal Complementary Therapies

**DOI:** 10.7759/cureus.46033

**Published:** 2023-09-26

**Authors:** Yash Kalariya, Ajay Kumar, Atta Ullah, Ahmed Umair, FNU Neha, FNU Madhurita, Vaidheesh Varagantiwar, Syed Muhammad Ibne Ali Jaffari, Arghal Ahmad, Mateen Aman, FNU Sapna, Giustino Varrassi, Satesh Kumar, Mahima Khatri

**Affiliations:** 1 Internal Medicine, Civil Hospital, Rajkot, IND; 2 Medicine, Jinnah Postgraduate Medical Center, Karachi, PAK; 3 Internal Medicine, Cavan General Hospital, Cavan, IRL; 4 Internal Medicine, Khyber Teaching Hospital (KTH), Peshawar, PAK; 5 Medicine, Fatima Memorial College of Medicine and Dentistry, Lahore, PAK; 6 Medicine, Jinnah Sindh Medical University, Karachi, PAK; 7 Medicine and Surgery, Shaheed Mohtarma Benazir Bhutto Medical University, Larkana, PAK; 8 Medicine, Rajiv Gandhi Institute of Medical Sciences, Adilabad, IND; 9 Medicine and Surgery, Shalamar Medical and Dental College, Lahore, PAK; 10 Medicine, Ziauddin University, Karachi, PAK; 11 Medicine, Shanxi Medical University, Jinzhong, CHN; 12 Pathology, Albert Einstein College of Medicine, Bronx, USA; 13 Pain Medicine, Paolo Procacci Foundation, Rome, ITA; 14 Medicine and Surgery, Shaheed Mohtarma Benazir Bhutto Medical College, Karachi, PAK; 15 Medicine and Surgery, Dow University of Health Sciences, Karachi, PAK

**Keywords:** gap, therapies, conventional, complementary, integrative, medicine

## Abstract

The global incidence of renal disorders is on the rise, demanding the implementation of novel and comprehensive strategies for patient care. The present study demonstrates the significance of renal health, offering a comprehensive comprehension of renal physiology and the escalating load of renal illnesses. The relevance of controlling renal illnesses is underscored by a thorough examination of conventional treatments, which encompass pharmaceutical interventions, dialysis, and transplantation. Subsequently, the story redirects its attention towards complementary therapies, classifying them into several categories, such as herbal medicine, acupuncture, dietary supplements, and mind-body activities, among various others. This paper presents a comprehensive examination of the available information, providing a critical study of the effectiveness and safety of alternative therapies in renal care. This study focuses on the central idea of integrative medicine, distinguished by its patient-centered and holistic approach and its seamless integration of conventional and complementary therapies. This study examines several integrative care models, using case studies to illustrate successful integrative approaches that have enhanced patient outcomes. The review thoroughly examines the current body of literature on integrative renal care, including meta-analyses, systematic reviews, and notable research discoveries. This study highlights the need for further research to address knowledge gaps and explore areas that require additional examination. These findings emphasize the importance of future research endeavors in this crucial sector. In addition, the paper thoroughly examines the safety issues and regulatory factors pertaining to complementary therapies, underscoring the importance of making educated decisions and maintaining diligent monitoring to safeguard patients' well-being. Integrating patient perspectives, experiences, and shared decision-making is essential to the integrated healthcare process, promoting a collaborative and patient-centered approach. The study culminates by providing a concise overview of the primary discoveries and delineating the ramifications of implementing therapeutic procedures. This statement underscores the considerable potential of integrative medicine in augmenting renal care, ultimately leading to enhanced patient outcomes and an improved overall quality of life for persons with renal diseases. Also, this literature review provides a thorough and knowledgeable examination of the incorporation of conventional and complementary therapies in the context of renal health. It gives valuable perspectives for healthcare practitioners, researchers, and policymakers interested in enhancing care strategies for individuals with renal conditions.

## Introduction and background

In the past several decades, the healthcare sector has undergone a notable transformation characterized by a shift towards a comprehensive and patient-centric approach. This transition has led to the emergence of the idea of integrated medicine [1]. Integrative medicine is characterized by amalgamating mainstream Western medicine with complementary and alternative therapies. It places significant emphasis on the holistic treatment of patients, taking into account not only their physical well-being but also their mental, emotional, and spiritual dimensions [2]. This technique has garnered significant attention and support throughout several medical disciplines. Within the realm of renal health, it exhibits substantial potential. The maintenance of renal health, which pertains to the overall well-being and optimal functioning of the kidneys, holds significant significance concerning persons' general health and quality of life [3]. Chronic kidney disease (CKD), kidney stones, and other renal disorders provide significant difficulties for patients and healthcare systems globally. Conventional medicine, equipped with various pharmacological interventions, dialysis, and transplantation, is crucial in resolving these concerns [4]. Nevertheless, conventional therapies frequently focus on particular facets of renal health, resulting in a deficiency in addressing individuals' overall well-being and quality of life. Integrating complementary and alternative therapies into renal care is a topic of interest and possible significance within the given context [5]. Complementary therapies comprise various disciplines, such as herbal therapy, acupuncture, dietary supplements, and mind-body interventions. When carefully combined with conventional treatments, these therapies can effectively meet the comprehensive needs of patients with renal conditions [6]. The integration described is consistent with the concepts of integrative medicine, which prioritizes a patient-centered approach that recognizes the distinctiveness of each individual and customizes treatment programs appropriately [6].

Incorporating both conventional and unconventional therapy within renal health should not be underestimated. Renal disorders frequently manifest various physical and psychological symptoms, encompassing pain, exhaustion, anxiety, and depression, which traditional treatments in isolation may not comprehensively alleviate [7]. In addition, the persistent nature of numerous renal disorders requires the implementation of extended care approaches that prioritize not only the treatment of the disease itself but also the improvement of patients' general well-being. In the past several decades, the healthcare sector has undergone a notable transformation characterized by a shift towards a comprehensive and patient-centric approach [8]. This transition has led to the emergence of the idea of integrated medicine. Integrative medicine is characterized by amalgamating mainstream Western medicine with complementary and alternative therapies. It places significant emphasis on the holistic treatment of patients, taking into account not only their physical well-being but also their mental, emotional, and spiritual dimensions [9]. This technique has garnered significant attention and support throughout several medical disciplines. Within the realm of renal health, it exhibits substantial potential. The maintenance of renal health, which pertains to the overall well-being and optimal functioning of the kidneys, holds significant significance concerning persons' general health and quality of life [7]. CKD, kidney stones, and other renal disorders provide significant difficulties for patients and healthcare systems globally. Conventional medicine, equipped with various pharmacological interventions, dialysis, and transplantation, is crucial in resolving these concerns [8]. Nevertheless, conventional therapies frequently focus on particular facets of renal health, resulting in a deficiency in addressing individuals' overall well-being and quality of life [9].

Integrating complementary and alternative therapies into renal care is a topic of interest and possible significance within the given context. Complementary therapies comprise various disciplines, such as herbal therapy, acupuncture, dietary supplements, and mind-body interventions [8]. When carefully combined with conventional treatments, these therapies can effectively meet the comprehensive needs of patients with renal conditions. The integration described is consistent with the concepts of integrative medicine, which prioritizes a patient-centered approach that recognizes the distinctiveness of each individual and customizes treatment programs appropriately [9]. Incorporating both conventional and unconventional therapy within renal health should not be underestimated. Renal disorders frequently manifest various physical and psychological symptoms, encompassing pain, exhaustion, anxiety, and depression, which traditional treatments in isolation may not comprehensively alleviate [10]. In addition, the persistent nature of numerous renal disorders requires the implementation of extended care approaches that prioritize not only the treatment of the disease itself but also the improvement of patients' general well-being. Incorporating complementary therapies can mitigate symptoms, diminish the adverse effects of traditional treatments, and augment patients' general well-being [11]. The utilization of this approach has the potential to enable patients to actively participate in making informed decisions regarding their healthcare, thereby cultivating a sense of autonomy and responsibility in managing their health trajectories [12]. Moreover, combining several components can enhance treatment adherence and ultimately improve outcomes.

The primary objective of this narrative review is to comprehensively examine, evaluate, and consolidate the current body of knowledge about integrative medicine strategies within the realm of renal health. Through an extensive review of scholarly literature, our objective is to thoroughly examine the idea of integrative medicine and its utilization within the context of renal care. By conducting a comprehensive analysis of research findings, case studies, and patient perspectives, our objective is to thoroughly understand the possible advantages, difficulties, and constraints related to incorporating complementary therapies into conventional treatments for renal conditions. Our objectives are diverse. Initially, the objective is to establish and elucidate the fundamental tenets of integrative medicine, emphasizing its patient-centered and comprehensive characteristics [9]. Furthermore, our objective is to classify and assess a range of supplementary therapies frequently employed in renal care to elucidate the current body of data substantiating their effectiveness and safety. Furthermore, our objective is to analyze and address the obstacles and difficulties that impede the smooth incorporation of complementary medicines into conventional renal healthcare. This includes an examination of safety concerns and regulatory factors [10]. In conclusion, we aim to underscore the crucial significance of patient perspectives, the practice of shared decision-making, and the imperative for additional research within this dynamic domain [11].

The primary aim of this narrative review is to furnish healthcare practitioners, researchers, and policymakers with an all-encompassing resource that clarifies the notion of integrative medicine and provides valuable perspectives on its implementation in the context of renal care [12]. The primary objective of this review is to provide a valuable contribution to the ongoing discourse on enhancing renal healthcare. The ultimate aim is to enhance the general welfare and quality of life for those affected by renal disorders [12]. Incorporating complementary therapies can mitigate symptoms, diminish the adverse effects of traditional treatments, and augment patients' general well-being [13]. The utilization of this approach has the potential to enable patients to actively participate in making informed decisions regarding their healthcare, thereby cultivating a sense of autonomy and responsibility in managing their health trajectories. Moreover, combining several components can enhance treatment adherence and ultimately improve outcomes [14].

The primary objective of this narrative review is to comprehensively examine, evaluate, and consolidate the current body of knowledge about integrative medicine strategies within the realm of renal health. Through an extensive review of scholarly literature, our objective is to thoroughly examine the idea of integrative medicine and its utilization within the context of renal care. By conducting a comprehensive analysis of research findings, case studies, and patient perspectives, our objective is to thoroughly understand the possible advantages, difficulties, and constraints related to incorporating complementary therapies into conventional treatments for renal conditions [3]. Initially, the objective is to establish and elucidate the fundamental tenets of integrative medicine, emphasizing its patient-centered and comprehensive characteristics [4]. Furthermore, our objective is to classify and assess a range of supplementary therapies frequently employed in renal care to elucidate the current body of data substantiating their effectiveness and safety [5]. Furthermore, our objective is to analyze and address the obstacles and difficulties that impede the smooth incorporation of complementary medicines into conventional renal healthcare. This includes an examination of safety concerns and regulatory factors [4]. In conclusion, we aim to underscore the crucial significance of patient perspectives, the practice of shared decision-making, and the imperative for additional research within this dynamic domain.

The primary aim of this narrative review is to furnish healthcare practitioners, researchers, and policymakers with an all-encompassing resource that clarifies the notion of integrative medicine and provides valuable perspectives on its implementation in the context of renal care. The primary objective of this review is to provide a valuable contribution to the ongoing discourse on enhancing renal healthcare. The ultimate aim is to enhance the general welfare and quality of life for those affected by renal disorders.

## Review

Renal health and conventional approaches

Maintaining renal health is crucial for optimal overall well-being, as the kidneys play a pivotal role as the principal filtration system in the human body [1]. The kidneys are essential organs that play a crucial role in maintaining homeostasis by filtering blood, controlling electrolyte levels, and regulating blood pressure [2]. This narrative review aims to comprehensively analyze the significance of renal health, examine prevalent kidney problems, and describe standard therapeutic approaches for managing renal disorders, including medicines, dialysis, and transplantation.

Importance of Renal Health

Maintaining renal health is essential for achieving and sustaining the balance within the body to support normal physiological activities [2]. The kidneys, a pair of renal organs located posteriorly in the abdominal cavity, provide a diverse range of functions fundamental to our overall well-being and lifespan [3].

Filtration and the elimination of toxins: A fundamental aspect of renal function is the remarkable process of filtering blood, which entails processing around 180 liters daily [4]. The process of filtration functions as an initial barrier, effectively removing metabolic waste products, excess salts, and pollutants [5]. The kidneys play a crucial role in preventing the buildup of detrimental substances that can potentially threaten our overall well-being by effectively filtering the bloodstream with great precision [6]. Fluid and electrolyte balance is a critical process regulated by the kidneys to maintain homeostasis inside the body [2-4]. The homeostatic control mechanisms effectively manage sodium, potassium, calcium, and phosphate concentrations, maintaining these essential ions within tightly controlled and vital ranges [3]. The presence of imbalances has the potential to result in the development of hypertension, cardiac arrhythmias, and neuromuscular abnormalities [4].

Blood pressure regulation: Blood pressure regulation is primarily controlled by the renin-angiotensin-aldosterone system, which is carefully regulated by the kidneys [5]. The kidneys have a crucial role in preventing hypertension and its associated problems, such as stroke and heart disease, through regulating blood volume and constricting blood vessels [6].

Erythropoiesis stimulation: The process of erythropoiesis stimulation involves the creation of erythropoietin, a hormone synthesized by the kidneys, which stimulates the generation of red blood cells within the bone marrow [2]. Sufficient quantities of red blood cells are crucial for the oxygenation of tissues, and the maintenance of renal health is closely associated with preserving this critical physiological activity [4].

Metabolism of vitamin: The metabolic process of vitamin D involves the conversion of its inactive form to its physiologically active form, which occurs primarily in the kidneys [7]. The metabolite mentioned above plays a crucial role in facilitating the absorption of calcium and phosphate in the intestines, which are vital constituents for maintaining optimal bone health [8].

Common Renal Conditions and Prevalence

Gaining knowledge about the spectrum of renal problems and their prevalence serves as a foundation for comprehending the implications of these disorders on the overall well-being of the population [9]. CKD is a prevalent condition defined by the progressive and slow decline in renal function [10]. The genesis of this condition is frequently associated with underlying health conditions, such as diabetes, hypertension, or glomerulonephritis. The prevalence of CKD globally is significant, impacting a large population of individuals with various severity levels [11]. If not adequately managed, CKD has the potential to progress to the point of renal failure, which would require interventions to preserve life [12].

Acute kidney injury (AKI): AKI refers to a sudden and potentially reversible deterioration in renal function, typically triggered by severe medical conditions, traumatic incidents, or the use of nephrotoxic agents [2]. Although AKI may be temporary, failure to promptly address it can lead to the development of CKD, highlighting the importance of rapid therapy [3]. Kidney stones, scientifically called renal calculi, are mineral concretions that develop within the kidney's tubules [4]. The transit of the urinary system might elicit intense pain. In recent years, there has been an observed increase in the incidence of kidney stones, which can be attributed to many dietary and lifestyle variables [5].

Polycystic kidney disease (PKD): PKD is a hereditary condition that follows an autosomal dominant inheritance pattern [6]. The development of many cysts within the renal system distinguishes it. Over some time, these cysts progressively increase in size, leading to the displacement of normal kidney tissue and hindering the appropriate functioning of the organ [7]. Although PKD is not commonly observed, it can profoundly impact individuals, perhaps influencing numerous generations within familial contexts [8].

Urinary tract illnesses (UTIs): Urinary tract infections are prevalent bacterial illnesses that have the potential to impact many components of the urinary system, encompassing the kidneys [9]. If left untreated, severe urinary tract infections (UTIs) can potentially progress into pyelonephritis, characterized by a kidney infection that can result in irreversible kidney damage [10].

Conventional Treatment Modalities for Renal Conditions

The management strategies for renal disorders are varied and individualized, with treatment options selected based on the specific nature of the sickness [11].

Regulation of blood pressure: The control of hypertension plays a pivotal role in providing healthcare for individuals with renal conditions [12]. Pharmaceutical agents such as Angiotensin-Converting Enzyme (ACE) inhibitors and Angiotensin II Receptor Blockers (ARBs) are commonly administered in clinical practice to reduce blood pressure and ameliorate renal burden [13]. In addition to regulating blood pressure, these medicines also provide renoprotective benefits.

Diuretics: Diuretics, often known as water pills, function to eliminate surplus fluid from the body, thereby alleviating the burden on the renal system [14]. Loop diuretics, such as furosemide, are frequently utilized to manage edema linked to renal failure.

Phosphate binders are frequently used in cases of CKD as they commonly lead to disturbances in phosphate levels [14]. Phosphate binders, such as sevelamer or calcium-based binders, are crucial in regulating serum phosphate levels, reducing the likelihood of developing secondary hyperparathyroidism and bone disease [15].

Pain management: Effective pain management is crucial for illnesses such as kidney stones. Nonsteroidal anti-inflammatory medicines (NSAIDs) and opioids are commonly recommended to alleviate the severe discomfort accompanying passing kidney stones [16].

Antibiotics are pivotal in managing urinary tract infections (UTIs) and kidney infections. Antibiotic choice depends on the particular infection and its susceptibility profile. The timely beginning of antibiotic therapy is crucial to prevent the advancement of infection in the renal system [17].

Dialysis is a medical procedure to remove waste products and excess fluids from the blood. Hemodialysis is considered an essential therapy for patients suffering from kidney failure, as it plays a crucial role in sustaining their lives. The process involves utilizing a dialysis machine to remove waste materials and excess fluids from the bloodstream [14]. During the hemodialysis process, the patient's blood is extracted from their body and directed via a dialyzer, where it undergoes purification before being reintroduced into the patient's circulatory system. Hemodialysis sessions are commonly scheduled multiple times each week within dedicated clinics [15]. , Peritoneal dialysis is a viable alternative to hemodialysis, offering the advantage of being a self-administered modality that may be conveniently performed in the comfort of one's home. The proposed approach leverages the abdominal cavity's peritoneum, a membranous lining, as an inherent filtration mechanism [15]. The process of peritoneal dialysis involves the introduction of dialysis fluid into the abdominal cavity, facilitating the absorption of waste products and excessive fluids. Following a period of residence, the fluid is subsequently evacuated, thereby facilitating the elimination of contaminants [16]. Continuous Renal replacement therapy (CRRT) is frequently utilized in managing critically ill individuals, particularly in intensive care settings. CRRT offers a method of waste product and fluid clearance characterized by a slower and more prolonged process, closely resembling the gradual filtration observed in the natural functioning of the kidneys [17]. This method offers several advantages in the management of hemodynamically unstable individuals.

Kidney transplantation: Kidney transplantation is widely regarded as the optimal approach for managing end-stage renal disease (ESRD), a critical phase characterized by a significant decline in kidney function [12]. In a kidney transplant procedure, a viable kidney sourced from a living or deceased donor is surgically transplanted into the recipient's body, thereby assuming the functional responsibilities of the compromised organ [13]. This intervention exhibits the most favorable potential for restoring a lifestyle that closely resembles normalcy, unencumbered by the limitations imposed by dialysis. Living donor transplants represent a feasible alternative where a willing and compatible donor is accessible. Typically, the persons who provide donations in such cases are frequently relatives, intimate acquaintances, or individuals driven by philanthropic motives [12]. Living donor transplants offer several benefits, such as reduced waiting periods and potentially improved outcomes resulting from the rapid accessibility of a healthy kidney [14]. Transplants from deceased donors pertain to the receipt of a kidney from a deceased individual who generously consented to organ donation [15]. The procedure above requires individuals to be placed on a waiting list because the accessibility of appropriate kidneys depends on a multifaceted interaction of variables, such as the availability of organs and the compatibility between donors and recipients [16]. After kidney transplantation, recipients must adhere to a lifelong regimen of immunosuppressive drugs. These pharmaceutical agents inhibit the immune system's ability to identify and mount an immunological response to the transplanted kidney, perceiving it as an external entity [17]. Immunosuppressive medicines, although essential for the survival of grafts, provide inherent hazards and require diligent monitoring.

Maintaining renal health is a crucial aspect of total physical and mental well-being, as the kidneys perform a wide range of intricate functions essential for sustaining life. Recognizing the importance of the kidneys, comprehending prevalent renal disorders, and acknowledging the diverse range of traditional therapeutic approaches such as medicines, dialysis, and transplantation are integral aspects of contemporary healthcare [18]. The increasing prevalence of renal disorders on a global scale emphasizes the necessity for ongoing research, innovation, and enhanced availability of treatments to protect renal health and promote the well-being of numerous individuals across the globe [19]. The interplay between medical knowledge, technological breakthroughs, and compassionate care greatly influences the preservation and restoration of renal health.

Complementary therapies in renal health

Complementary therapies have garnered considerable interest in renal health, presenting viable supplementary methods to traditional treatments. These therapeutic approaches comprise various interventions, such as herbal therapy, acupuncture, nutritional supplements, and other modalities [20]. This study aims to examine complementary treatments for renal health thoroughly. It will involve the establishment of clear definitions and classifications for these therapies, an in-depth analysis of the current data supporting their effectiveness in renal care, and a critical discussion of the obstacles and constraints connected with their implementation [21].

Complementary Therapies in the Context of Renal Health

Complementary treatments, commonly known as complementary and alternative medicine (CAM), cover a range of healthcare practices and procedures that go beyond the purview of orthodox Western medicine [22]. Within the realm of renal health, the medicines mentioned above can be roughly classified into subsequent domains.

Herbal medicine: Herbal remedies encompass the utilization of botanical ingredients for medicinal intentions. In renal care, there has been much investigation into the potential benefits of certain herbs, including astragalus, dandelion root, and nettle leaf, regarding their ability to enhance kidney function, mitigate inflammation, and alleviate symptoms commonly associated with renal disorders [22].

Acupuncture: Acupuncture, an integral aspect of traditional Chinese medicine (TCM), encompasses the precise insertion of slender needles into specific anatomical locations to facilitate the circulation of energy and the recovery process [23]. Certain acupuncture advocates propose that treatment could mitigate symptoms such as pain, nausea, and weariness frequently encountered by patients afflicted with renal disease [24].

Dietary supplements refer to various substances, including vitamins, minerals, and other chemicals commonly ingested as capsules, pills, or liquids [25]. The potential of supplements, such as coenzyme Q10, omega-3 fatty acids, and other antioxidants, to alleviate oxidative stress and inflammation has been explored in renal health. Mind-body practices encompass a range of activities, such as yoga, meditation, and tai chi, which emphasize the interrelation between mental and physical well-being [26]. These methods are occasionally employed to mitigate stress, promote general welfare, and optimize the management of chronic ailments, especially those about renal health [27].

TCM is a holistic treatment system encompassing several therapeutic approaches, such as acupuncture, herbal medicine, food therapy, and other techniques. TCM is frequently employed comprehensively within renal health to manage the manifestations and fundamental etiology of renal problems effectively [28]. Homeopathy is a therapeutic approach employing extensively diluted medicines to elicit a response from the body's healing capabilities. Certain persons who suffer from kidney diseases may opt for homeopathic medicines to alleviate specific symptoms or enhance their general well-being [29].

Effectiveness of Complementary Therapies in Renal Care

The effectiveness of complementary therapies in renal care is a topic that continues to be investigated and discussed, given the wide range of available interventions [30]. The available evidence about the utilization of various therapeutic interventions can be categorized in the following manner [31].

Herbal medicine: Certain herbal medicines have demonstrated the potential to enhance kidney health. One example is astragalus, a botanical commonly employed in TCM, which has been the subject of scientific investigation on its potential efficacy in mitigating proteinuria (abnormal levels of protein in urine) and enhancing glomerular filtration rates among individuals afflicted with CKD [12]. Likewise, dandelion root and nettle leaf may possess diuretic attributes, potentially conferring advantages to renal performance. Nevertheless, it is imperative to show prudence when utilizing herbal therapy, given that not all herbal therapies have proven safe or well-investigated for their efficacy in treating renal ailments [32]. The available research regarding the efficacy of acupuncture in renal treatment is inconclusive. Several studies have indicated that acupuncture could relieve symptoms often experienced by persons with kidney illness, including pain, exhaustion, and nausea [33]. However, it is essential to note that the quality of research conducted in this field exhibits inconsistencies, necessitating the implementation of more rigorous trials to conclusively determine the efficacy of acupuncture in treating these symptoms [34].

Dietary supplements: Specific dietary supplements have attracted considerable attention due to their possible advantages for renal health. One area of research that has garnered attention is the examination of omega-3 fatty acids due to their possible anti-inflammatory characteristics and ability to potentially mitigate cardiovascular risk factors in persons diagnosed with CKD [33]. The ability of Coenzyme Q10, an antioxidant, to alleviate oxidative stress in renal tissues has also been investigated. Nevertheless, adopting a cautious approach while considering nutritional supplements is imperative, as their excessive or incorrect utilization can result in unfavorable consequences [34].

Mind-body techniques, such as yoga and meditation, have effectively mitigated stress and enhanced general well-being. Consequently, these practices hold potential benefits for those afflicted with kidney disease [35]. Stress management holds significant importance in renal care, given that the persistence of chronic stress can worsen symptoms and compromise kidney function [36]. Although these behaviors may not provide direct treatment for the underlying renal problem, they can contribute to a more comprehensive approach to healthcare [37].

TCM has garnered significant attention for its comprehensive approach to managing renal diseases. Several studies have indicated that TCM therapies, such as herbal medicine and acupuncture, can potentially decrease proteinuria, enhance kidney function, and alleviate symptoms in persons diagnosed with CKD [38]. Nonetheless, the wide range of therapeutic approaches within TCM and the imperative for thorough scientific investigation underscores the significance of seeking guidance from trained practitioners [39].

Homeopathy involves the administration of personalized remedies that are specifically suited to address particular symptoms [40]. Although some individuals claim to have seen favorable outcomes when using homeopathy to manage symptoms related to renal diseases, it is essential to note that the available research supporting these claims is primarily based on anecdotal reports. Comprehensive evaluation of the efficacy of homeopathic remedies for renal health necessitates the implementation of well-designed clinical trials [2].

Challenges and Limitations

The incorporation of complementary therapies into the field of renal care has many obstacles and constraints. One of the main obstacles in renal care is the insufficiency of robust scientific evidence that substantiates the effectiveness of numerous supplementary therapies [23]. Although several medicines exhibit potential, conducting more rigorous research, such as randomized controlled trials, is imperative to confirm their efficacy conclusively. Numerous complementary therapies, including herbal medicines and dietary supplements, possess the capacity to interact with drugs and elicit potential adverse effects [22]. Individuals afflicted with renal disorders need to seek guidance from healthcare practitioners who possess expertise in these treatments to guarantee their safe and appropriate utilization. One notable issue within complementary therapies is the absence of standardization, particularly about dosages, treatment procedures, and practitioner training [24]. The variety of these products can provide difficulties in reliably evaluating their effectiveness and safety. The cost and accessibility of specific complementary therapies might provide challenges, as these interventions can often be associated with high expenses and limited insurance coverage. Furthermore, there may be limited availability of proficient practitioners specializing in specific therapeutic modalities, such as acupuncture or TCM, in some geographical regions [34].

Variability among individuals: The responses to complementary therapies exhibit considerable variation across individuals. The efficacy of a particular approach may vary among individuals. Individual variability highlights the significance of implementing individualized methods in healthcare [22].

Integration with traditional care: Complementary therapies must be utilized in conjunction with, rather than as a substitute for, traditional medical treatments to manage renal problems [12]. The attainment of a cohesive integration between these two methodologies can present challenges and necessitates proficient communication among patients, healthcare professionals, and practitioners of complementary therapies [13].

In conclusion, it can be concluded that complementary therapies present a wide range of interventions that possess the capacity to augment renal care. Although certain medicines can potentially enhance renal health and mitigate symptoms, further thorough investigation is necessary to confirm their usefulness conclusively [14]. Furthermore, it is crucial to emphasize the significance of informed decision-making and collaboration between patients and healthcare professionals in renal care when contemplating the utilization of complementary therapies. This is due to safety concerns, the absence of standardization, and individual variability [15]. In conclusion, an integrative strategy that synergistically incorporates the merits of alternative and traditional medicine has the potential to provide the most all-encompassing healthcare for those afflicted with renal disorders.

Concept and principles of integrative medicine

Integrative medicine is an approach to healthcare that prioritizes the needs and preferences of patients, aiming to integrate mainstream Western medicine with complementary or alternative therapies to optimize outcomes [16]. Fundamentally, integrated medicine acknowledges the intricate and interrelated nature of the human body, adopting a comprehensive viewpoint that incorporates all aspects of well-being, including physical, emotional, mental, and spiritual dimensions. Several fundamental principles support this technique. Patient-centered care is fundamental to integrative medicine, as it prioritizes the patient's role and involvement in their healthcare journey [14]. The approach underscores a collaborative relationship between the healthcare practitioner and the individual receiving care, placing importance on the patient's choices, values, and objectives. Integrative care involves the active engagement of patients in their health decision-making processes, with a particular emphasis on carefully considering their individualized needs and experiences. The holistic approach of integrative medicine recognizes that health and wellness encompasses more than simply the absence of disease [16]. The perspective acknowledges the holistic nature of the individual, acknowledging the interdependence of the physical, mental, and spiritual aspects. A holistic approach in healthcare prompts healthcare providers to consider several dimensions of a patient's existence, encompassing their lifestyle choices, environmental factors, and emotional state [17]. Integrative medicine aims to integrate the merits of conventional Western medicine with evidence-based complementary therapies. Instead of perceiving these treatments as mutually exclusive, integrative care aims to combine them to synergistically offer comprehensive and tailored treatment programs [18].

The emphasis on prevention is a fundamental principle within integrative medicine. The focus is on implementing tactics to uphold and enhance health before the onset of sickness [18]. The proactive approach frequently encompasses lifestyle modifications, nutritional interventions, stress management techniques, and other preventive measures customized to meet the specific needs of the individual [19]. Integrative medicine lays significant importance on the utilization of evidence-based practices. To assure the safety and effectiveness of complementary therapies, they must be substantiated by scientific studies and clinical data [20].

Justification for the Integration of Conventional and Complementary Approaches in Healthcare

Various persuasive justifications support the incorporation of conventional and complementary approaches in healthcare. Integrative medicine can enhance patient outcomes by offering improved results. Integrating the advantageous aspects of conventional and complementary therapies facilitates a more all-encompassing and individualized approach to healthcare [12]. This phenomenon can result in improved management of symptoms, heightened quality of life, and superior overall patient health outcomes. Treatment customization is a crucial aspect of healthcare as it recognizes the inherent uniqueness of each individual and acknowledges the variability in their healthcare demands. Integrative medicine facilitates the customization of treatment programs by healthcare practitioners to cater to individual patient's unique requirements and inclinations [13]. The customization process has the potential to yield medicines that exhibit enhanced efficacy and improved tolerability. The mitigation of side effects is a common concern in conventional medical treatments, as these treatments might challenge certain patients [14]. Integrative care can encompass complementary therapies that effectively alleviate the adverse effects associated with treatment, enhancing the overall treatment experience [15].

The comprehensive approach of integrative medicine aims to discover and address the fundamental causes of illness rather than solely focusing on symptom management. This methodology has the potential to result in enhanced sustainability and enduring enhancements in health [16]. Promoting wellness and prevention is a primary focus within integrative medicine, which places significant importance on proactive healthcare measures and lifestyle adjustments. The likelihood of chronic diseases can be mitigated by facilitating patients in making healthier choices and embracing preventative steps, hence fostering holistic well-being [17]. Patient satisfaction is frequently attributed to integrative medicine's personalized and patient-centric approach. The experience of being listened to and actively participating in one's healthcare can result in increased levels of patient satisfaction and engagement [18].

Patient-Centered and Holistic Approach of Integrative Medicine

Integrative medicine's concept and practice are oriented around patients' holistic care. Within integrative medicine, the patient assumes a role that extends beyond being a passive recipient of care, instead becoming an engaged and collaborative participant in their healthcare trajectory [2]. The healthcare professional dedicates effort towards comprehending the patient's values, preferences, and goals, guaranteeing that treatment plans meet the patient's unique requirements. The concept of holistic assessment is rooted in the recognition of integrative medicine, which posits that the state of health is influenced by a multifaceted interplay of several aspects, including but not limited to the physical, emotional, mental, and spiritual dimensions [3]. Integrative healthcare specialists provide comprehensive evaluations that encompass all aspects of the patient's well-being, including their medical background, lifestyle choices, sources of stress, emotional state, and personal belief systems [4]. The field of integrative medicine acknowledges the variability in treatment outcomes among individuals, emphasizing the need for personalized approaches. The development of treatment plans is characterized by a high degree of individualization, wherein conventional therapies are integrated as needed, and evidence-based complementary therapies are incorporated when deemed suitable [4]. Customizing treatments guarantees that patients are provided with the most efficacious and comprehensive care.

Integrative medicine lays a significant focus on preventative care and health promotion. Patients are advised to actively preserve their well-being by implementing lifestyle changes, adhering to proper nutrition, managing stress levels, and adopting other preventive strategies [5]. This proactive approach is consistent in preserving general well-being. The field of integrative medicine recognizes the significant correlation between the mind and body. In healthcare, it is common to incorporate various practices, such as meditation, yoga, and mindfulness, into treatment protocols to target emotional and psychological well-being effectively [6]. The practice of integrative medicine frequently entails the adoption of a collaborative methodology, wherein healthcare professionals from diverse fields collaborate harmoniously to deliver all-encompassing treatment [7]. Collaboration across different disciplines can comprehensively comprehend the patient's requirements and enhance efficacy in devising therapeutic approaches.

In summary, integrative medicine embodies a patient-centric, comprehensive, and empirically grounded methodology for healthcare that amalgamates the merits of mainstream Western medicine and complementary therapies [8]. Integrative medicine can enhance patient outcomes and promote overall well-being by customizing treatment regimens to individual requirements, prioritizing prevention, and emphasizing the mind-body link [9]. The statement highlights a significant change in the healthcare field, as there is a growing acknowledgment of the need to address the entirety of an individual's well-being rather than solely focusing on the ailment [10]. This approach also emphasizes the empowerment of patients, enabling them to actively participate in managing their health.

Integrative approaches in renal health

Recently, there has been a notable increase in the recognition and use of integrative medicine, which emphasizes a comprehensive and patient-centric methodology that integrates mainstream Western medicine with complementary and alternative therapies [11]. This methodology has demonstrated potential in diverse medical disciplines, encompassing renal health. Integrative approaches in renal treatment strive to comprehensively address the multifaceted dimensions of patients' well-being, extending beyond the physical manifestations of renal problems to embrace their emotional, psychological, and spiritual requirements [12]. This section delves into various models of integrative care that can be employed in renal health. Additionally, it presents case studies and examples that demonstrate the efficacy of integrative approaches in this domain [13].

Collaborative treatment teams: A prevailing approach to integrative treatment in renal health entails forming collaborative care teams consisting of healthcare professionals representing many disciplines. The composition of these teams frequently includes several healthcare professionals such as nephrologists, nurses, nutritionists, social workers, and complementary therapists, among others [14]. Implementing a multidisciplinary approach facilitates effective communication and collaboration among team members, hence facilitating a thorough assessment of patients' requirements [15]. Collaborative care teams can develop individualized treatment plans that integrate conventional treatments, dietary guidelines, stress reduction methods, and additional complementary therapies. This strategy guarantees that patients receive comprehensive care that encompasses their medical symptoms and their emotional and psychological well-being [15].

Patient-centered care: Patient-centered care is a core idea within integrative medicine and is of particular significance in renal health. The significance of engaging patients in treatment decisions and customizing care regimens to align with their needs and preferences is underscored. Integrative renal care prioritizes patient-centered healthcare, enabling patients to engage actively in the decision-making process regarding their treatment choices [17]. This strategy frequently involves engaging in shared decision-making dialogues between patients and healthcare practitioners, wherein patients are provided with information regarding both conventional and complementary therapies, enabling them to make well-informed decisions that align with their personal beliefs and objectives [18].

Using case studies and examples is common in academic research and analysis. These tools illustrate and support theoretical concepts, providing real-world applications and empirical evidence. By examining specific instances or scenarios. A patient, aged 50, who has been diagnosed with CKD, sought medical attention at an integrative healthcare center [19]. The patient's treatment regimen consisted of pharmacotherapy and routine dialysis sessions. To mitigate the patient's deteriorating quality of life and psychological anguish, the integrative care team integrated mindfulness-based stress reduction (MBSR) strategies, dietary counseling, and acupuncture into the therapy regimen [20]. Over the course of the study, the patient consistently reported a decrease in anxiety levels and an enhancement in their general well-being. Furthermore, the implementation of dietary modifications by the patient played a role in improving blood pressure regulation, thereby highlighting the possible synergistic effects that might arise from the integration of conventional and alternative strategies in managing CKD [21].

The individual suffering from recurring nephrolithiasis had difficulties associated with persistent discomfort and anxiety. In conjunction with conventional medical treatments, the patient chose to pursue integrative therapy, encompassing the utilization of herbal remedies, modifications to food patterns, and the implementation of relaxation techniques. During the treatment, the patient documented a notable decrease in the severity of pain experienced and the frequency of kidney stone occurrences [22]. The incorporation of these complementary therapies not only effectively alleviated the patient's pain but also significantly enhanced their overall quality of life [22-24].

A kidney transplant recipient was involved in a pilot initiative that integrated mind-body techniques, including yoga and meditation, into the post-transplantation rehabilitation protocol. The techniques above were implemented to mitigate stress, enhance the quality of sleep, and promote the patient's general mental and emotional well-being [13-16]. The patient's compliance with immunosuppressive drugs demonstrated a notable improvement, resulting in a reported enhancement in the patient's quality of life compared to non-participants of the program [18]. This case underscores the potential advantages of incorporating mind-body techniques alongside traditional therapy within kidney transplantation. The presented case studies and examples demonstrate the potential of integrative approaches in renal health, highlighting their ability to enhance both clinical outcomes and the overall quality of life for patients [20]. By customizing treatment programs based on individual requirements and integrating complementary therapies, healthcare practitioners have the potential to augment the comprehensive care provided to persons with renal diseases. Integrative care models promote interprofessional collaboration and patient empowerment, facilitating a comprehensive and patient-centric approach to renal health [21].

Evidence-based research on integrative approaches in renal health

Evidence-based research has grown interest in utilizing integrative techniques for promoting renal health. These techniques aim to integrate mainstream Western medicine with complementary or alternative therapies to offer a more comprehensive and holistic approach to treating kidney-related disorders [32-35]. An examination of the current scholarly literature indicates an increasing amount of research investigating the effectiveness and possible advantages of integrative approaches in renal care. Several meta-analyses and systematic reviews have been conducted to investigate the effects of integrative approaches on renal health. A noteworthy meta-analysis was conducted to evaluate the impact of acupuncture as a supplemental intervention for individuals with CKD. The present study, published in the “Journal of Clinical Medicine” in 2020, conducted a systematic review and meta-analysis of various randomized controlled trials (RCTs) [30]. This analysis revealed that the adjunctive use of acupuncture with conventional treatments yielded noteworthy enhancements in renal function parameters, including glomerular filtration rate (GFR) and serum creatinine levels.

Using herbal medicine to treat CKD has garnered significant attention in research endeavors. In 2021, a systematic study was published in the journal “Evidence-Based Complementary and Alternative Medicine” that investigated the efficacy of herbal remedies, such as astragalus and nettle leaf, as prospective supplementary treatments for managing CKD [31]. The research emphasized that several herbal therapies exhibited the potential to mitigate proteinuria and impede the advancement of CKD. Nevertheless, the authors placed significant emphasis on the necessity for additional rigorous research to establish the effectiveness of the interventions definitively [32]. The literature has extensively investigated the effects of mind-body practices on stress reduction and enhancement of quality of life in patients diagnosed with kidney disease. In 2019, a notable study was published in the journal “Psychoneuroendocrinology” that examined the impact of MBSR on individuals receiving hemodialysis treatment [33]. The study's results suggest that participation in MBSR may be associated with a decrease in symptoms related to depression and anxiety, an improvement in the quality of sleep, and a reduction in the feeling of pain [33]. These studies highlight the potential of mind-body techniques in improving the overall well-being of individuals with renal conditions.

Limitations in Existing Research and Areas Requiring Additional Exploration

The extant body of literature about integrative approaches in renal health offers valuable insights [34]. Yet, it is evident that several gaps and areas warrant further investigation. The presence of variation in integrative therapies presents a significant obstacle in reaching conclusive findings, necessitating the establishment of standardized protocols. Establishing standardized protocols for the delivery of complementary therapies is necessary to achieve uniformity and reproducibility in research investigations [35].

Long-term consequences: Numerous studies primarily examine short-term outcomes, resulting in a scarcity of data about the enduring impacts of integrative approaches in renal treatment. Investigating the enduring advantages and possible hazards linked to these therapeutic interventions over prolonged durations is imperative. Combination therapies frequently encompass the utilization of numerous complementary therapies alongside conventional treatments in integrative approaches [23]. Further study is warranted to evaluate these combination therapies' synergistic effects and safety profiles. Evaluating cost-effectiveness is crucial in assessing the value of integrative techniques, especially within healthcare systems that face resource constraints [33]. There is a need for comparative studies that assess the expenses and the benefits. The power of integrative medicine resides in its capacity to customize treatments according to individualized requirements, leading to personalized medicine [34]. Nevertheless, the current state of study regarding individualized techniques and predicted indicators for therapy response is still in its early stages and necessitates additional inquiry [35].

Safety and unwanted effects: Although numerous complementary therapies are usually regarded as safe, it is crucial to thoroughly assess any potential unwanted effects and interactions with conventional pharmaceuticals. The preservation of patient safety remains of utmost importance [36].

Patient-reported outcomes (PROs): Integrative medicine lays a significant emphasis on providing care centered around the patient's needs and preferences [37]. It is imperative for ongoing research to integrate PROs to evaluate the effects of these interventions on several aspects, such as quality of life, symptom management, and general well-being [23].

In summary, evidence-based research on integrative approaches in renal health is continuously growing, providing valuable insights into their possible advantages. Prominent meta-analyses, systematic reviews, and empirical investigations have provided insights into the effectiveness of integrative therapies in enhancing renal function, alleviating symptoms, and improving the quality of life among individuals with renal conditions [23]. Nevertheless, the existing body of research has several deficiencies that underscore the necessity for more inquiry. These deficiencies include the absence of standardized protocols, the lack of long-term outcome assessments, and the dearth of cost-effectiveness evaluations. Consequently, it is imperative to continue investigating these areas [24]. The potential for the future of renal treatment lies in the patient-centered and customized approach of integrative medicine, as long as research endeavors focus on addressing existing gaps and establishing robust evidence-based practice [40].

Safety and regulatory considerations

The safety of complementary therapies is paramount due to the diverse range of interventions involved, such as herbal remedies, acupuncture, dietary supplements, and mind-body activities. Although numerous complementary therapies are typically considered safe when taken correctly, addressing the potential side effects and safety issues associated with these treatments is essential [12]. The utilization of herbal treatments has the potential to result in adverse outcomes or diminished effectiveness of drugs due to possible interactions [13]. One example is St. John's wort, a frequently utilized herbal supplement, which has the potential to impede the efficacy of specific prescription treatments. Moreover, several herbs may elicit adverse effects, including gastrointestinal disruptions or hypersensitivity reactions. Acupuncture is considered a safe therapeutic intervention when administered by a skilled and certified practitioner [15]. Nonetheless, failure to adhere to appropriate cleanliness practices and needle insertion techniques may result in a negligible probability of infection or damage [16].

Dietary supplements, encompassing vitamins and minerals, can be deemed safe when consumed following suggested dosages. Nevertheless, consuming excessive specific dietary supplements might result in toxicity and detrimental impacts on an individual's overall well-being [17]. Mind-body techniques, such as meditation and yoga, are typically regarded as safe and well-tolerated. However, it is essential to note that persons with specific medical conditions, such as severe musculoskeletal disorders, may encounter discomfort or worsening symptoms if they are not provided with appropriate instructions or guidance [18]. These safety issues emphasize The need to seek guidance from competent healthcare professionals with expertise in complementary medicines. Patients must disclose to their healthcare professionals any complementary therapies they are currently utilizing or contemplating to handle potential interactions or dangers effectively [19].

Regulatory Landscape Surrounding Complementary Therapies

The regulatory framework about complementary therapies exhibits considerable variation among countries and regions, rendering it intricate owing to the multifarious nature of methods and products encompassed within this domain. Regulatory approaches can be broadly classified into various categories [20]. Certain complementary therapies have been subjected to regulatory measures, establishing them as distinct healthcare professions. In numerous jurisdictions, licensing, and regulation are in place to govern chiropractors, acupuncturists, and naturopathic doctors, thereby guaranteeing that these practitioners adhere to prescribed education and training criteria [21]. Dietary supplements, encompassing vitamins, minerals, and herbal items, are subject to regulatory oversight as food products in certain jurisdictions. The regulatory rules for dietary supplements in the United States were created by the Dietary Supplement Health and Education Act (DSHEA) of 1994 [22]. Nevertheless, the level of regulatory scrutiny for non-pharmaceutical products may be less stringent.

The regulations about herbal medications might exhibit significant variation. In several nations, herbal products are subjected to regulatory measures like those imposed on pharmaceuticals, necessitating compliance with established safety and efficacy criteria [23]. In alternative contexts, these products might be classified as dietary supplements, subject to less stringent regulations. Consumer protection is a prevalent concern in numerous nations, leading to the implementation of regulatory measures aimed at safeguarding customers against deceptive or inaccurate assertions about complementary therapies [24]. One illustration of this is the monitoring and enforcement of truth-in-advertising legislation by advertising standards authorities. Professional associations play a crucial role in guiding complementary therapy practices by establishing and enforcing standards of practice, codes of ethics, and requirements for ongoing education among practitioners [25].

Patient awareness of the regulatory environment within their respective regions and their proactive pursuit of trained practitioners who conform to established treatment standards are crucial [26]. Furthermore, healthcare providers must be knowledgeable about the regulatory status of complementary therapies to offer appropriate advice and safeguard the safety of their patients.

Interactions and Conflicts With Conventional Therapies

The integration of complementary therapies with traditional medical treatments has the potential to generate significant advantages. Yet, it also gives rise to concerns regarding potential interactions or conflicts. Several crucial factors need to be taken into account [27]. Certain complementary therapies, specifically herbal medicines and dietary supplements, have the potential to interact with prescribed medications. For example, herbal supplements such as St. John's wort, garlic, or ginkgo biloba can disrupt specific medications' metabolic processes, impacting their efficacy and safety [22]. Healthcare providers must perform comprehensive medication evaluations and diligently monitor potential drug interactions.

Contraindications: There are certain medical situations where specific complementary therapies may not be recommended [25]. For instance, it is essential to note that certain herbs may provide potential risks for persons who have liver disease or kidney failure. Acupuncture may present contraindications for patients who have bleeding issues or are currently using blood-thinning drugs [27].

Treatment plans: To mitigate potential conflicts, healthcare providers must communicate transparently with patients regarding their utilization of complementary therapies [35]. The principle of shared decision-making should guide the integration of complementary therapies into a patient's treatment plan. It is imperative to consider the patient's preferences, goals, and values, as this contributes to a comprehensive understanding of their healthcare needs. Employing a collaborative approach in the decision-making process is crucial to optimize the overall outcomes and benefits for the patient [35].

In conclusion, it is imperative to prioritize the safety and adherence to regulatory standards of complementary therapies to promote patients' overall well-being. Patients and healthcare professionals must collaborate to manage complementary medicines' intricate nature effectively [6]. This collaboration should encompass a comprehensive understanding of potential adverse effects, regulatory implications, and the potential interactions that may arise with traditional treatments. Utilizing a collaborative and well-informed approach facilitates the achievement of a harmonious equilibrium between the advantages offered by complementary therapies and the safeguarding of patients within the framework of conventional healthcare [9].

Role of Patient Perspectives and Shared Decision-Making in the Context of Integrative Medicine

Gaining insight into patients' viewpoints on integrative techniques is crucial to delivering care based on the patient's needs and preferences. Many individuals resort to complementary therapies for diverse purposes, and their encounters can provide significant perspectives. Themes commonly observed in patient experiences include the amelioration of overall well-being, the augmentation of symptom control, and a heightened sense of self-efficacy in managing their health [10]. For example, individuals receiving cancer treatment may choose complementary therapies such as acupuncture or meditation to mitigate the adverse effects associated with treatment, ease stress, and restore a sense of agency. Individuals suffering from chronic pain often seek alternative therapeutic approaches such as chiropractic care or massage therapy to enhance their overall well-being and decrease their need for pain drugs [12]. Patient testimonies also highlight the significance of individualized healthcare. Patients highly value healthcare practitioners who allocate sufficient time to listen to their concerns, preferences, and objectives actively [13]. The organization places high importance on practitioners who actively participate in shared decision-making and work collaboratively to create treatment programs consistent with the values and beliefs of the individuals involved [14].

Patient Preferences and Motivations for Selecting Complementary Therapies

The reasons for patients in selecting complementary therapies are diverse and can encompass several factors. Complementary therapies are often favored by numerous patients due to their holistic nature, which encompasses the physical, emotional, and spiritual aspects of health [16]. Individuals prefer therapeutic interventions that target the underlying causes of their ailments rather than exclusively concentrating on alleviating symptoms. Complementary therapies commonly exhibit a lower incidence of side effects than traditional treatments. Patients may undergo these therapeutic interventions to mitigate the negative consequences commonly associated with pharmaceuticals or invasive medical procedures [18]. Using complementary therapies can enable patients to assume an active and participatory role in their healthcare. Practices such as mindfulness meditation and dietary adjustments provide individuals with practical strategies for managing their diseases and enhancing their general well-being. Patients desire personalized care that acknowledges and honors their distinct requirements and preferences [19]. Complementary therapies frequently provide individualized treatment regimens, enabling patients to customize their care following their unique values and lifestyles [20].

Improved quality of life: A significant number of patients opt for complementary therapies as a means to enhance their overall quality of life [23]. Various therapeutic techniques, such as massage therapy and art therapy, have been found to have a positive impact on mental well-being, stress reduction, and general life satisfaction [24].

Significance of Collaborative Decision-Making

The establishment of shared decision-making between patients and healthcare providers holds the utmost importance within the framework of integrative medicine [31]. The process above guarantees that treatment plans are congruent with the patient's preferences and objectives while also considering evidence-based procedures and safety measures. The process of shared decision-making is initiated by establishing transparent and compassionate communication. Healthcare practitioners must actively listen when patients express their worries and share their experiences with complementary therapies [32]. In doing so, healthcare providers should understand and recognize patients' intentions and expectations. Furthermore, the practice of shared decision-making encompasses a comprehensive evaluation of the potential dangers and advantages associated with complementary medicines and their potential interactions with conventional treatments [22]. Healthcare practitioners are crucial in delivering evidence-based information to inform patients' decision-making processes and safeguard their well-being.

In summary, patients' viewpoints on integrative techniques underscore the significance of individualized healthcare and the various reasons for opting for complementary therapies [23]. The concept of shared decision-making between patients and healthcare practitioners recognizes the need to consider several views, granting individuals the agency to engage in their healthcare actively and guaranteeing that treatment plans are based on comprehensive information and following patients' desires and objectives [26]. Patient-centered care in integrative medicine acknowledges and upholds the comprehensive aspect of individuals' well-being while promoting a cooperative method toward achieving optimal health.

Challenges and future directions in integrating complementary therapies into conventional healthcare

Obstacles to the Integration of Complementary Therapies

The incorporation of complementary therapies into conventional healthcare holds significant potential for enhancing patient-centered care and addressing a diverse array of health issues. Nevertheless, some obstacles hinder the smooth integration of new therapies into conventional healthcare systems. One main obstacle in alternative therapies is the absence of standardized practices [25]. In contrast to traditional medicine, which adheres to defined protocols and norms, complementary therapies comprise a wide range of activities, generally characterized by varied levels of regulation and standardization [29]. The absence of standardization in this context may perplex healthcare professionals and provide challenges in establishing coherent, evidence-driven protocols. Despite an increasing volume of scholarly investigations on complementary therapies, numerous interventions continue to exhibit a dearth of substantial scientific data substantiating their effectiveness and safety [40]. Integrating alternative therapies into traditional healthcare presents a notable barrier due to the emphasis on evidence-based practice as the prevailing standard. Healthcare practitioners may exhibit reluctance to integrate therapies lacking a robust empirical data foundation [33].

The regulatory and legal obstacles to complementary medicines vary significantly across diverse areas and countries. Specific complementary therapies may face a dearth of well-defined laws, posing difficulties in guaranteeing the safety and quality of these modalities [34]. Furthermore, the integration process may encounter obstacles due to legal and liability concerns, as there is apprehension around the possibility of malpractice lawsuits arising from unfavorable outcomes. Establishing effective communication and collaboration among healthcare practitioners is crucial in facilitating the successful integration of complementary therapies [31]. Nonetheless, communication gaps and a dearth of shared comprehension between traditional healthcare physicians and practitioners of complementary therapy can impede effective collaboration. Misunderstandings and skepticism might arise due to variations in vocabulary, therapeutic approaches, and educational backgrounds. One of the critical challenges in incorporating complementary therapies is the presence of financial constraints, which encompass various expenses such as practitioner fees, equipment, and training. Numerous healthcare systems and insurance carriers lack coverage for these expenses, presenting financial difficulties for both healthcare facilities and patients [35]. A financial obstacle may impede individuals' ability to avail themselves of alternative therapies.

Communication Challenges Among Healthcare Providers

Establishing effective communication among healthcare providers is paramount to delivering complete and coordinated care that encompasses complementary therapies [22]. Nevertheless, various obstacles can hinder effective collaboration. Conventional healthcare doctors and alternative therapy practitioners frequently exhibit contrasting philosophical orientations regarding patient care. Conventional medicine typically adheres to a reductionist paradigm, prioritizing examining and treating individual diseases and symptoms [23]. In contrast, complementary therapies frequently adopt a holistic and patient-centric standpoint. Navigating these divergent philosophical perspectives and establishing a shared foundation for collaborative efforts can provide significant difficulties [35].

Education and awareness: Many healthcare providers may have a restricted understanding and awareness regarding complementary therapies. The current emphasis in medical school frequently places conventional treatments at the forefront, resulting in a lack of preparedness among healthcare providers to engage in discussions or make recommendations regarding alternative medicines [24]. The absence of comprehension in this context may result in skepticism and hesitancy to provide referrals for supplementary therapy services. One potential issue within healthcare systems is the absence of established communication mechanisms for facilitating communication and referrals between conventional healthcare professionals and complementary healthcare providers [11]. Fragmented care, missed opportunities for collaboration, and uncertainty about roles and responsibilities can be potential outcomes of this situation.

Patient safety concerns: Healthcare professionals may possess valid apprehensions regarding the safety and effectiveness of some complementary therapies, mainly when employed alongside traditional treatments. These issues can hinder effective communication and collaboration among healthcare providers, as their primary focus is ensuring patient safety and minimizing the risk of injury [12]. Healthcare personnel frequently operate within bustling clinical environments characterized by restricted time availability for communication and collaboration. The task of allocating sufficient time for discussing and integrating complementary therapies can present difficulties, hence compounding the existing communication gaps [11].

Prospects for Future Research and Approaches to Addressing Obstacles

The successful integration of complementary medicines into conventional healthcare necessitates a comprehensive strategy that spans various dimensions, including research, education, policy, and collaboration [15]. The imperative for future research lies in producing robust evidence substantiating alternative therapies' safety and effectiveness. Utilizing well-structured clinical trials, systematic reviews, and meta-analyses can contribute significantly to establishing the efficacy of specific interventions and developing a more robust body of evidence [16].

Standardization and training: Implementing standardized training and certification programs for complementary therapy practitioners can augment their qualifications and promote uniformity in their practice [19]. Healthcare organizations can emphasize the recruitment of highly skilled practitioners and offer continuous professional development opportunities for their personnel. The integration of complementary therapy education into the healthcare curriculum has the potential to foster mutual understanding and collaboration among healthcare providers, hence promoting interprofessional education [20]. Interprofessional training programs have the potential to enhance communication and teamwork among healthcare professionals, promoting a more comprehensive and cohesive approach to delivering patient care. Formulating evidence-based clinical practice guidelines pertaining to incorporating complementary medicines might provide healthcare practitioners with valuable direction [21]. These guidelines determine the appropriate timing and methodology for integrating complementary therapies into patient care, focusing on maintaining both safety and efficacy.

Implementing a patient-centered strategy, which actively engages patients in the decision-making process about complementary therapies, is of utmost importance [22]. Patients must be provided with reliable and precise information, are motivated to engage in open discussions regarding their preferences and objectives with their healthcare providers, and are actively involved in shared decision-making [24]. Integrating complementary therapies within healthcare systems can be effectively facilitated by creating well-defined communication channels and fostering collaborative relationships between conventional healthcare professionals and complementary therapy practitioners [36]. This may entail establishing interdisciplinary teams, using electronic health record systems that enhance information exchange, and establishing networks for patient referrals. Advocacy endeavors may exert influence over health policy and reimbursement frameworks to facilitate the incorporation of complementary therapies [37]. Implementing measures such as broadening insurance coverage for specific therapies and promoting collaboration among healthcare providers can mitigate financial obstacles and foster the exploration of complementary treatment choices when deemed suitable [38].

In summary, successfully integrating complementary medicines into conventional healthcare necessitates a collaborative endeavor encompassing research, education, legislative modifications, and enhanced interprofessional communication within the healthcare community [39]. By adopting a patient-centered approach and adhering to evidence-based practice, healthcare providers can effectively address these obstacles and deliver comprehensive, holistic treatment that caters to patients' varied requirements and preferences [40].

## Conclusions

In conclusion, this narrative review has thoroughly explored the complex terrain of incorporating conventional and unconventional medicines within renal care. The key findings highlight the increasing evidence supporting the effectiveness of specific complementary therapies, including acupuncture, herbal therapy, and mind-body activities, in enhancing renal health. Nevertheless, several obstacles must be overcome to achieve a smooth integration, such as standardization, communication gaps between healthcare providers, and regulatory restrictions. The combination of these factors holds considerable implications for clinical practice, underscoring the significance of patient-centered, holistic care that customizes treatment programs according to each individual's unique requirements and preferences. Through the cultivation of collaboration and the facilitation of shared decision-making, this method has the potential to improve patient outcomes, mitigate adverse effects, and bolster overall well-being within renal care. In conclusion, incorporating both conventional and complementary medicines presents an opportunity to enhance the standard of renal care, indicating a hopeful trajectory for the future of healthcare within this field.
